# Fullerenes’ Interactions with Plasma Membranes: Insight from the MD Simulations

**DOI:** 10.3390/biom12050639

**Published:** 2022-04-26

**Authors:** Nililla Nisoh, Viwan Jarerattanachat, Mikko Karttunen, Jirasak Wong-ekkabut

**Affiliations:** 1 Department of Physics, Faculty of Science, Kasetsart University, Bangkok 10900, Thailand; nililla.ni@ku.th; 2 Computational Biomodelling Laboratory for Agricultural Science and Technology (CBLAST), Faculty of Science, Kasetsart University, Bangkok 10900, Thailand; viwan.jar@nectec.or.th; 3 Thailand Center of Excellence in Physics (ThEP Center), Ministry of Higher Education, Science, Research and Innovation, Bangkok 10400, Thailand; 4 NSTDA Supercomputer Center (ThaiSC), National Electronics and Computer Technology Center (NECTEC), National Science and Technology Development Agency (NSTDA), Pathumthani 12120, Thailand; 5 Department of Chemistry, The University of Western Ontario, 1151 Richmond Street, London, ON N6A 3K7, Canada; mkarttu@uwo.ca; 6 Department of Physics and Astronomy, The University of Western Ontario, 1151 Richmond Street, London, ON N6A 3K7, Canada; 7 The Centre for Advanced Materials Research (CAMBR), The University of Western Ontario, 1151 Richmond Street, London, ON N6A 3K7, Canada

**Keywords:** coarse-grained molecular dynamic simulations, fullerenes, plasma membrane, carbon nanoparticle

## Abstract

Understanding the interactions between carbon nanoparticles (CNPs) and biological membranes is critically important for applications of CNPs in biomedicine and toxicology. Due to the complexity and diversity of the systems, most molecular simulation studies have focused on the interactions of CNPs and single component bilayers. In this work, we performed coarse-grained molecular dynamic (CGMD) simulations to investigate the behaviors of fullerenes in the presence of multiple lipid components in the plasma membranes with varying fullerene concentrations. Our results reveal that fullerenes can spontaneously penetrate the plasma membrane. Interestingly, fullerenes prefer to locate themselves in the region of the highly unsaturated lipids that are enriched in the inner leaflet of the plasma membrane. This causes fullerene aggregation even at low concentrations. When increasing fullerene concentrations, the fullerene clusters grow, and budding may emerge at the inner leaflet of the plasma membrane. Our findings suggest by tuning the lipid composition, fullerenes can be loaded deeply inside the plasma membrane, which can be useful for designing drug carrier liposomes. Moreover, the mechanisms of how fullerenes perturb multicomponent cell membranes and how they directly enter the cell are proposed. These insights can help to determine fullerene toxicity in living cells.

## 1. Introduction

Fullerenes (C60) have attracted a lot of attention since their discovery in 1985 [1]. Their unique electronic properties together with biocompatibility [2,3,4], effective permeation and hydrophobic properties make them suitable for applications in nanomedicine, especially for drug delivery, bioimaging, photodynamic therapy, and as anticancer agents [5,6,7,8,9].

Despite the broad range of applications and growing use, there are some rather severe problems. One particularly important problem is the damage fullerenes cause to biological membranes. The physical origin of this, despite being critical knowledge for biological applications, remains unclear [10]. Several experimental studies have investigated the toxicity of C60 [11,12,13]. It is known that due to fullerenes’ low solubility in water, C60 molecules aggregate causing health and environmental hazards [14,15,16,17]: Sayes et al. [14] have demonstrated that fullerene aggregates induce oxidative stress, have strong toxicity, and produce adverse effects in fish, human skin, liver cells and lung tissue. Porter et al. [18] used electron microscopy and showed that C60 aggregates are present even in the cytoplasm, lysosomes and, especially, in cell nuclei. Additionally, at high concentrations, C60-induced vesicle rupture, membrane leakage and cell death have been demonstrated [19,20,21].

Despite the abundance of studies demonstrating cell membrane damage, the physical mechanisms of how C60 causes cell damage are not well understood. Molecular dynamics (MD) simulations have offered some insights. Results from simulations have suggested that the effects of fullerenes depend on concentration and lipid types, in particular the degree of acyl chain saturation and chain length [22,23,24,25,26,27]. Some controversies also exist. One particular example is that no membrane damage has been directly observed in simulations even at high concentrations; only changes in mechanical properties of membranes have been reported [22,23,25,26,28]. One notable exception are the simulations by Xie et al., which reported that large C60 aggregations cause mechanical damage to the membrane based on the observation that reverse micelles emerged [27]. In addition, and perhaps even more surprisingly, some experiments did not observe any C60 aggregation [29,30].

To date, most studies have focused on fullerenes in single-lipid species bilayers [27,30,31] or binary mixtures [26]. However, real cell membranes are complex multicomponent systems. The eukaryotic plasma membrane (PM) contains hundreds of different lipid types, varying amounts of cholesterol, and the two leaflets (inner and outer) have asymmetric compositions [32,33]. Such complexity makes the plasma membrane difficult to study both experimentally and computationally. Therefore, understanding the interactions between fullerenes and realistic cell membranes is challenging, yet it is imperative in being able to determine the exposure risk in the living organisms at atomic resolution. In this study, we used CGMD simulations to study the interactions of fullerenes (C60) with a multicomponent plasma membrane. The mechanisms of how nanoparticles interact with cell membranes and where the fullerenes locate in real cell membranes are investigated to reduce adverse effects on living beings.

## 2. Methodology

### 2.1. Unbiased Molecular Dynamics Simulations

The initial structure of the plasma membrane was taken from the simulations of Ingólfsson et al. [34]. The model consisted of 63 different lipid types, including 14 lipid headgroups and 11 lipid tails asymmetrically distributed between the two leaflets. Of the lipid headgroups, phosphatidylserine (PS), phosphatidic acid (PA), phosphatidylinositol (PI), phosphatidylinositol phosphates (PIPs) and lysophosphatidylcholine (LPC) were in the inner leaflet, whereas glycolipids (GM), ceramide (CER), diacylglycerol (DAG)were in the outer leaflet. Phosphatidylcholine (PC), phosphatidylethanolamine (PE), and sphingomyelin (SM) are present in both leaflets. All lipid tails were represented by 3–6 tail beads. The plasma membranes were composed of ~6600 lipids, 30 mol% cholesterol, ~2000 Na^+^, and ~1200 Cl^−^ ions in a 40 × 40 × 13 nm^3^ box. Each system was solvated with 124,216 water molecules. (Tables S1 and S2) show the numbers of each lipid type and mole fractions in the two leaflets of a plasma membrane. To study membrane-fullerene interactions, 314, 629, 1363, and 1992 fullerene molecules, corresponding to concentrations of 5%, 10%, 20%, and 30%, respectively, were added.

The force field parameters in this work are based on Ingólfsson et al. [34] who used the Martini version 2.1 [35] coarse-grained (CG) model for lipids, water, ions, and modifying a few lipids. The membrane structures were generated by the INSANE (INSert membrANE) tool [36]. Further details of the plasma membrane can be found in ref. [34]. The Martini version 2.1 [37] force field was used for the fullerene model with 16 beads. Two methods were used to add fullerenes into membranes: (1) all fullerenes were initially placed closed to the lipid headgroups in the water phase, and they spontaneously penetrated the membranes and (2) fullerenes were pulled into the membrane with a constant force pulling 1500 kJ mol^−1^nm^−2^.

Simulations were performed with the GROMACS software package v5.1.2 [38] under the NPT ensemble. A semi-isotropic pressure coupling of 1 bar was maintained with the Parrinello–Rahman barostat [39] with a time constant of 5.0 ps and compressibility of 3×10^-4^ bar^−1^. The temperature of 298 K was controlled by the Parrinello–Donadio–Bussi velocity rescaling thermostat [40] with a time constant of 1.0 ps. Non-bonded interactions were calculated based on the parameters of ref. [41], where electrostatic interactions were calculated using the reaction field method with a relative dielectric constant of ***ε***_r_ = 15 and shifted to zero at the cutoff of 1.2 nm. The Lennard-Jones interactions were shifted to zero at the cutoff distance of 1.2 nm using the potential-shift-Verlet modifier method. The time step of simulations was kept at 20 fs. All systems were simulated for 10 µs, and the last 5 µs were used for the analysis. Periodic boundary conditions were applied to all systems. The Visual Molecular Dynamics (VMD) software version 1.9.3 [42] was used for all visualizations. The MDAnalysis python package [43,44] was employed for correlation and density analyses.

### 2.2. Biased Molecular Dynamics Simulations

The numbers of molecules, parameters, and conditions were identical to those in unbiased simulations. In the biased simulations, all fullerenes (fullerene concentrations of 5%, 10%, 20%, and 30%) were initially placed above the upper membrane at ~10 nm from the center of the plasma membrane. The fullerenes rapidly formed a large cluster in the water phase and were then pulled to the membrane interior. After 45 ns, the fullerenes were located inside the membrane. A force constant of 1500 kJ mol^−1^ nm^−2^ was used for biasing potential in the z-direction (membrane normal). All simulations were equilibrated for 10 µs. The last 5 µs were used for analysis. For the high concentrations of fullerenes (20% and 30%), the large cluster of aggregated fullerenes in water could damage membranes before entrance. Therefore, we performed additional simulations using biased systems by gradually pulling fullerenes into the plasma membrane one-by-one until saturation was reached at the concentration of 19% (1296 fullerenes) and 204,216 water. The dynamics of fullerenes in the neat plasma membrane at high fullerene concentrations were investigated.

### 2.3. Calculation of Potential of Mean Force Profiles 

To study energetics, umbrella sampling simulations with the weighted histogram analysis method [45,46] (WHAM) were performed. The pulling direction was along the membrane normal (z-axis). The simulated bilayers contained 512 lipid molecules and 16,000 CG water beads. Fullerene concentrations of 0% and 40% and DAPC were used. To calculate the potential of mean force (PMF), a single fullerene was pulled from the water phase (z = 4.5 nm) to the center of the bilayer (z = 0 nm) using steps of 0.1 nm. A fullerene was restrained by a harmonic constant of 1000 kJ mol^−1^. The systems were simulated in the NVT ensemble at 298 K with a simulation time of 1 µs per each umbrella window (the total time was 46 µs). For histograms, the boot-strapping analysis method was used [46].

## 3. Results and Discussion

### 3.1. Fullerene Translocation in Asymmetric Plasma Membrane

**Figure 1:**
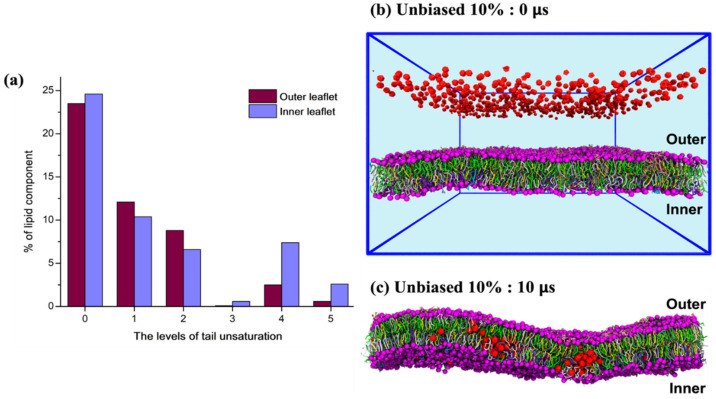
(**a**) The distribution of lipid tail unsaturation in the inner and outer leaflets of a plasma membrane. The lipid compositions are reported in Table S3. Snapshots from an unbiased simulation at 10% fullerene concentration (**b**) at the beginning (0 µs) and (**c**) after 10 µs. Fullerenes are shown in red, phosphate groups in purple, and cholesterols in yellow. Lipids are colored by their headgroups type (PC: green, PE: white, SM: cyan, GM: pink, PS: violet, PIPs: aqua, other: ice blue).

The plasma membrane is asymmetric [32,47]. The lipid distributions of the different headgroups are displayed in Figure S1. The inner leaflet typically consists of lipids with four or five levels of tail unsaturation more than the outer leaflet (Figure 1a). To investigate the role of the asymmetric lipid distribution on fullerene–membrane interactions, we placed fullerenes in the water phase at 10 nm (Figure 1b) from the outer leaflet and performed unbiased MD simulations for 10 µs (see Methodology for more details). Similarly to previous studies that used single-lipid bilayers [27,31,48], independent of the studied concentration (5%, 10%, 20%, and 30%), fullerenes rapidly aggregated to small clusters in water and spontaneously penetrated into the plasma membrane. Afterward, they stably located inside the plasma membrane. Interestingly, fullerenes preferred to be in the inner leaflet rather than the outer one (Figure 1c). In addition, membrane damage was observed at 20% and 30% concentrations (Figure S2). Table 1 summarizes the membrane structures and C60 aggregation at the end of the simulations.

### 3.2. Fullerenes Prefer the Inner Leaflet

**Figure 2:**
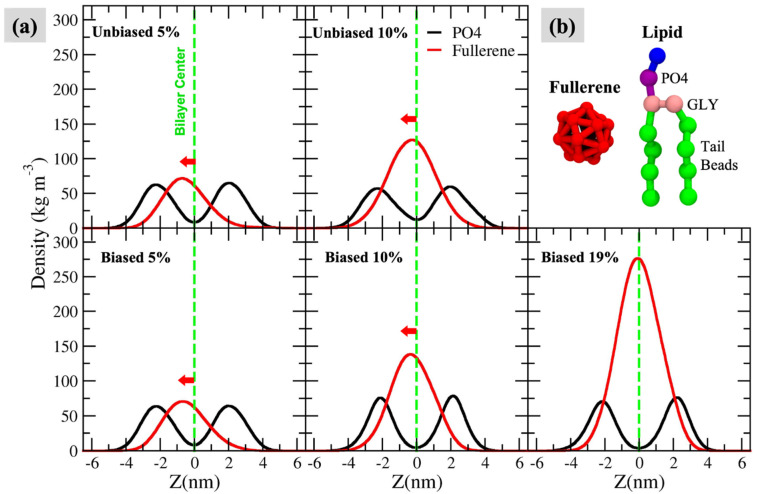
(**a**) Density distribution profiles of the phosphate groups (PO4 beads) and fullerenes in the z-direction for biased and unbiased simulations at fullerene concentrations of 5%, 10%, and 19%. The dashed lines represent the bilayer center (z = 0 nm). The data of biased and unbiased simulations with fullerene concentrations of 20% and 30% are not presented because the plasma membranes were unable to maintain the bilayer structure. (**b**) The MARTINI coarse-grained (CG) models of fullerene and lipid.

Figure 2 and Figure S5 show the density distributions of fullerenes and the phosphate groups. In the 5% and 10% systems, the peak of fullerene densities shifted toward the inner leaflet. To investigate if this behavior is real and not an artifact from periodic boundary condition (PBC), a set of biased simulations with a biasing force of 1500 kJ mol^−1^nm^−1^ to pull fullerenes from water toward the outer leaflet was performed. The results in Figure 2 also show that the inner leaflet is the preferred location. However, when fullerene concentration was increased, the density peak shifted toward the bilayer center. A possible explanation of this behavior is that at high concentrations the space in the inner leaflet was not enough to accommodate the C60 molecules, resulting in a symmetrical distribution of fullerenes in both leaflets. Note that the plasma membranes could not maintain the bilayer structure at 20% and 30% concentrations (Figure S2).

### 3.3. The Relations of Fullerene Location and Lipid Saturation Levels

In the previous section, we established that the fullerenes penetrate the membrane and interact with lipid tails rather than lipid headgroups, in agreement with previous studies [22,23]. Moreover, the preferred location of fullerenes is at the inner leaflet in the regions that have elevated levels of tail unsaturation. In plasma membranes, the inner leaflets have typically higher levels of unsaturation than the outer leaflets (Figure 2).

**Figure 3:**
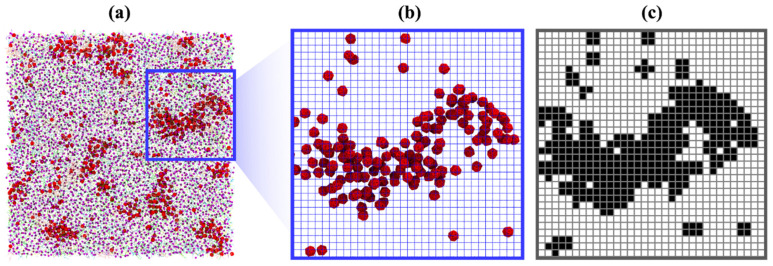
(**a**) Top view of the system; fullerenes are shown in red. The blue square indicates the enlarged region. (**b**) Top view of the enlarged region showing the grid. Molecules other than fullerenes are not shown for clarity. (**c**) The corresponding ‘shadow’ matrix. Black (assigned the value of 1) means there is at least one fullerene in the grid space; white (the value of 0) means there is no fullerene present in the grid space.

**Figure 4:**
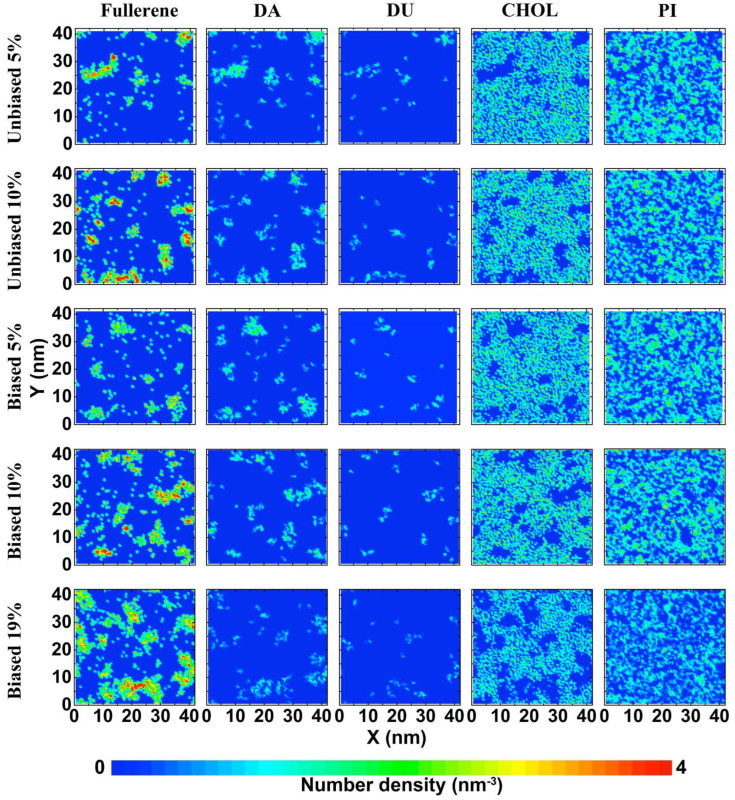
2D density maps of fullerenes, cholesterol, and the lipid tails (DA, DU, PI) in biased and unbiased simulations at fullerene concentrations of 5%, 10%, and 19%. Each density plot after 10 µs of simulation. The density maps are colored by relative enrichment (red) or depletion (blue).

To understand how lipid saturation levels affect the location of fullerenes, we analyzed correlations between the fullerenes using their locations in the x-y plane and the location of each type of lipid tail. To perform this analysis, the simulation box was gridded with spacing of 0.5 × 0.5 nm^2^. The molecules were then mapped onto this grid. Next, a ‘shadow’ matrix representing the location of the given molecule was generated; an example is shown in Figure 3. Then, the mean square error (MSE) was used to compare the similarity of the shadow matrixes between fullerenes and each type of lipid tail. Since fullerenes were located near the center of the bilayer, MSE was calculated considering both leaflets together. Low MSE value means that similarity is high, whereas high MSE value means that similarity is low. The MSE value of zero implies that the locations of the two given molecules are identical. Table 2 shows the average MSE values over the last 5 µs. The MSE calculation revealed that the interactions between fullerenes and DA tails are the most favorable among all lipid types, whereas fullerene and cholesterol are the least favored combination. These results agree with the density map (Figure 4 and Figure S4), in which the region of fullerene matches with the region of DA tails, whereas the regions of cholesterol and fullerenes are mismatched. Of the top five high preferential interactions, fullerenes localize in the region of polyunsaturated lipids (four and five double bonds). Considering the top five high ranked MSE values, we found that fullerenes avoided the regions populated by cholesterol and mono-, di-unsaturated lipids. This suggests that the saturation level in the tail lipid group is the most likely factor that guides fullerene localization in the plasma membrane. In addition, we also performed this analysis for the head group, see Figure S5. It was found that the fullerenes preferred to stay with the PE headgroups. However, headgroup preference appears to be a minor effect because DAPE is one of the lipids with both a PE headgroup with high tail unsaturation (DA tails).

### 3.4. Potential of Mean Force Profiles for Fullerene Translocation into Bilayers

**Figure 5:**
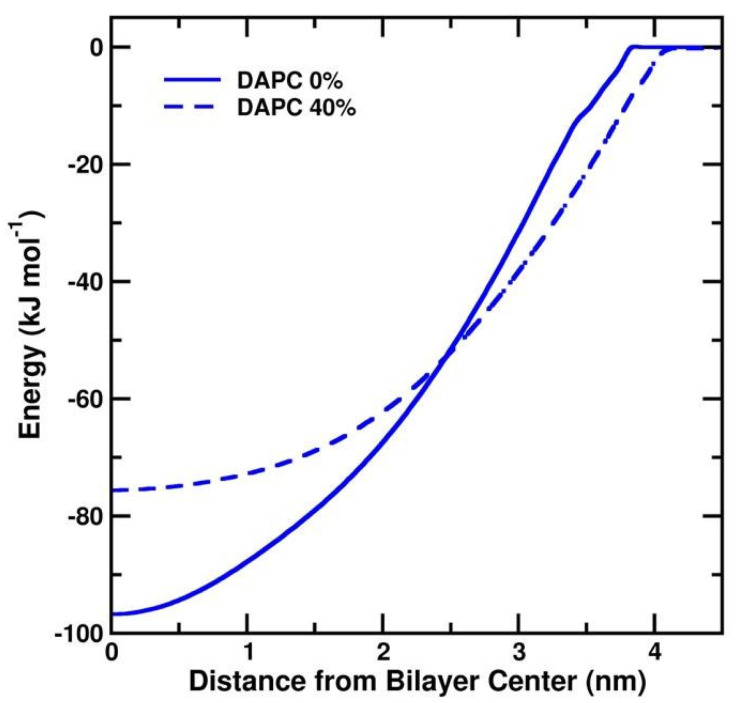
PMF profiles for moving a fullerene across DAPC lipid bilayers at fullerene concentrations of 0% (solid lines) and 40% (dashed lines).

To explain why fullerene prefers to stay with the DA tails, we calculated the potential of mean force profiles (PMF) of a single fullerene translocating across the highly unsaturated DAPC bilayer and compared the results with PMFs obtained for DLPC, DSPC, and DFPC bilayers from our previous study [22]. The PMF profiles show that the depth of minimum free energy depends on the type of the lipid tail. As shown in Figure 5, the minima in DAPC 0% and DAPC 40% are at about −96.7 ± 1.7 and −75.6 ± 0.6 kJ mol^−1^, respectively, when fullerenes are at the bilayer center. The PMF profiles of a fullerene in a DAPC bilayer are similar to the ones in DFPC (see the PMF in Figure S6). The minimum energies are at −94.4 ± 1.6 kJ and −73.1 ± 0.9 kJ mol^−1^ for DFPC 0% and DFPC 40% bilayers, respectively. Compared with the profiles in DLPC and DSPC, the free energy barriers for a fullerene are at −78.9 ± 0.9 and −83.8 ± 0.4 kJ mol^−1^ in DLPC 0% and DSPC 0%, respectively. The positions of the energy minima from the bilayer center are ~0.7 and ~1.4 nm for DLPC 0% and DSPC 0%, respectively.

### 3.5. Fullerene Aggregation and the Plasma Membrane Damage

In the modeled plasma membranes, fullerenes aggregated even at the lowest concentration (5%), and the clusters preferred to stay with unsaturated lipids. These observations are clearly different from what has been reported in previous studies using only single-lipid type bilayers. In those studies, fullerenes only aggregated at high concentrations (> 20%) and in saturated bilayers [22]. The PMF profiles can be used to explain these differences: In the homogeneous single-lipid type bilayer, at the certain z-position, the minimum energies at all positions in the bilayer plane are about the same (Figure S6). Therefore, fullerenes distribute in the middle of the bilayer or in the tail region and start to aggregate when the free space runs out. In contrast, for the plasma membrane, the energies are different depending on the lipid type, and fullerenes migrate to the regions rich in DAPC. Compared with the full size of the bilayer, these DAPC regions are relatively small, but they allow fullerene aggregation even at low concentrations. This explains not only why the results from the two simulation studies are different, but also why many experiments and simulations yield different results.

**Figure 6:**
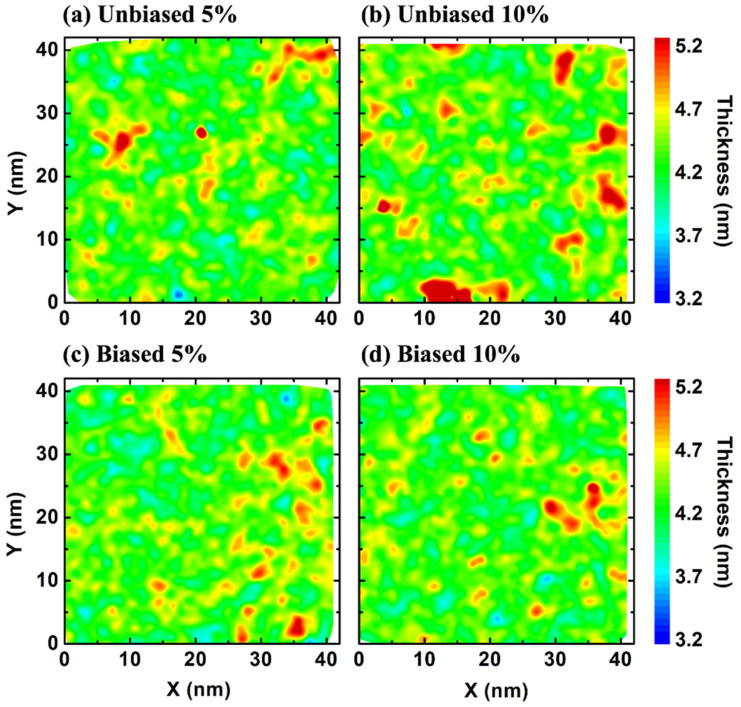
Bilayer thickness at (**a**) unbiased 5%, (**b**) unbiased 10%, (**c**) biased 5% and (**d**) biased 10% fullerene concentrations. Increase in plasma membrane thickness is correlated with the locations of fullerene aggregation. The titles in each of the figures show the fullerene concentration and if the simulation was biased or unbiased.

**Figure 7:**
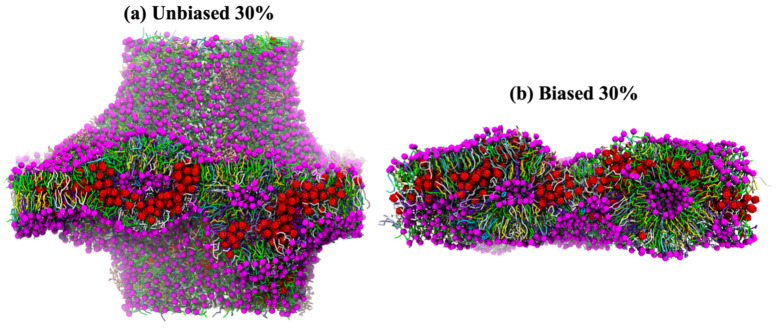
Plasma membrane damage. Side view of (**a**) unbiased and (**b**) biased simulations at fullerene concentration of 30%. Fullerenes are shown in red, cholesterol is colored yellow, and lipid headgroups are colored by type (PC: green, PE: white, SM: cyan, GM: pink, PS: violet, PIPs: aqua, other: ice blue).

To understand the effect of fullerene aggregation in the plasma membrane, we calculated the local thickness (Figure 6). The result shows that local thicknesses are correlated with the locations of fullerene aggregation. At lower concentration, fullerenes did not induce significant structural changes in plasma membrane, nonetheless they induced undulations in the structures (Figure S2). However, at high concentrations, two different behaviors in different situations were observed. First, in the unbiased simulations, fullerenes aggregated into large clusters in water and entered the membrane resulting in membrane deformation (Figure 7a). This behavior also was observed in a previous simulation study [27]. Second, in the biased simulation, fullerenes accumulated in the membrane and formed a large cluster. As a result, this induced some of the lipids to form vesicles inside of the bilayer (Figure 7b). Interestingly, this vesicle did not destroy the bilayer structure. However, the presence of a fullerene cluster and vesicle might perturb the transportation of small molecules resulting in loss of membrane function.

## 4. Conclusions

In this work, we investigated the interactions of fullerenes with asymmetric plasma membranes using coarse-grained molecular dynamics simulations. The results show that fullerenes form clusters even at low concentrations (5%) and prefer to locate in the inner leaflet rather than the outer one; fullerenes migrated from the water phase, moved through the outer leaflet, and finally populated the region of the inner leaflet. In the inner leaflet, fullerenes preferred to be aggregated in the tail regions of the DAPE lipids. The potential of mean force (PMF) was used to analyze the origin of this specificity. The results show that compared with other lipids, the minimum free energy of a fullerene in DAPC bilayer is the lowest and that the fullerenes favor to stay in the regions of DA-tail lipids, which are highly unsaturated. At low concentrations, the fullerene clusters caused membrane undulations but did not directly damage the plasma membrane. However, at high concentrations, membrane damage was observed in both unbiased and biased simulations. Our results also explain the fullerene aggregation mechanism which aggregates at low fullerene concentration and is only revealed when studying the fullerene behavior in plasma membrane. This supports the discovery of fullerene aggregations in the plasma membrane found by using an electron microscope [18]. This study provides new insights about fullerene toxicity in the complex membrane.

## Figures and Tables

**Table 1:** Summary of membrane structures and aggregation of fullerenes at the end of the simulations.

System	Concentration of Fullerene (%)	Plasma Membrane Structure	Aggregation
1	0%	Bilayer	-
2	Unbiased 5%	Undulation	Cluster
3	Unbiased 10%	Undulation	Cluster
4	Unbiased 20%	Membrane deformation	Cluster
5	Unbiased 30%	Membrane deformation	Cluster
6	Biased 5%	Undulation	Cluster
7	Biased 10%	Undulation	Cluster
8	Biased 19%	Undulation	Cluster
9	Biased 20%	Vesicle in membrane	Cluster
10	Biased 30%	Vesicle in membrane	Cluster

**Note:** Undulation: bilayer with large fluctuations, membrane deformation: membranes could not maintain bilayer structure, vesicle in membrane: some lipids incorporate by bending their polar head groups and remaining adhered to the membrane surface. Corresponding figures are shown in Figure S2.

**Table 2:** Mean square error (MSE) values comparing the average fullerenes and each type of lipid tail location for the inner and outer leaflets together over the last 5 µs.

Unbiased 5%		Biased 5%		Unbiased 10%		Biased 10%	
**Top 5 Smallest**							
DAPE DUPE PUPC DAPS DAPC	0.005 0.021 0.024 0.024 0.028	DAPE DUPE DAPC PUPC PUPS	0.004 0.015 0.024 0.025 0.026	DAPE PAPC PUPE DUPE PUPS	0.010 0.023 0.030 0.037 0.039	DAPE PAPC PUPE PUPS DUPE	0.017 0.022 0.026 0.041 0.042
**Top 5 Largest**							
CHOL PIPC PIPX DPSM POPE	0.406 0.183 0.174 0.158 0.143	CHOL PIPX PIPC POPX POPC	0.367 0.185 0.154 0.149 0.121	CHOL POPX PIPC PIPX POPE	0.350 0.150 0.144 0.123 0.117	CHOL POPX DPSM PIPX PNSM	0.308 0.138 0.127 0.117 0.110

**Note:** PO4 beads of PIPC and POPC version Ingólfsson [34] were restrained on the z-axis (renamed by PIPX and POPX). Our simulations had no restraints.

## Data Availability

Not applicable.

## Supplementary Materials

The following supporting information can be downloaded at: https://www.mdpi.com/article/10.3390/biom12050639/s1, Table S1: System details. Production simulations were run for 10 µs; Table S2: The compositions of lipids in the plasma membrane models; Table S3: Plasma membrane lipid tail distributions; Figure S1: Pie charts showing the distribution of the main lipid headgroups in the plasma membrane. Cholesterol is colored yellow and lipid headgroups are colored by type (PC: green, PE: white, SM: cyan, GM: pink, PS: violet, PIPs: aqua, other: ice blue); Figure S2: Side views at the end of the simulations at different fullerene concentrations in unbiased and biased simulations. Water molecules and ions are not shown for clarity. Red spheres: fullerenes molecules. Purple spheres: phosphate groups. Lipid colors are shown in Figure S1; Figure S3: Top views at the end of the simulations at different fullerene concentrations in unbiased and biased simulations of plasma membranes. Water molecules and ions are not shown for clarity. Red spheres: fullerenes molecules. Purple spheres: phosphate groups. Lipid colors are shown in Figure S1; Figure S4: Snapshots illustrating the preference of fullerenes to localize close to the DA tail lipids in biased and unbiased simulations at fullerene concentrations of 5% and 10%. Fullerenes are shown in red, phosphate groups in purple, and DA tails in white; Figure S5: 2D density map of fullerenes, lipid head groups (PE, PC, PS, SM) in biased and unbiased simulations at fullerene concentrations of 5%, 10%, and 19%. Each density plot after 10 μs of simulation. The density maps are colored by relative enrichment (red) or depletion (blue); Figure S6: PMF profiles for moving a fullerene across DLPC, DSPC, DFPC and DAPC lipid bilayers at fullerene concentrations of 0% (solid lines) and 40% (dashed lines).
