# Supplementary material for: Fullerenes’ Interactions with Plasma Membranes: Insight from the MD Simulations
Source: Biomolecules. 2022 Apr 26;12(5):639. doi: 10.3390/biom12050639 (PMC9138838; doi:10.3390/biom12050639)
Supplement: Supplementary file 1 [file biomolecules-12-00639-s001.zip › biomolecules-1662767-supplementary.pdf]

Fullerenes' Interactions with Plasma Membranes: Insight from the MD Simulations

Nililla Nisoh [1,2,3], Viwan Jarerattanachat [2,4], Mikko Karttunen [5,6,7] and Jirasak Wong-ekkabut [1,2,3,*]

[1] Department of Physics, Faculty of Science, Kasetsart University, Bangkok 10900, Thailand; nililla.ni@ku.th (N.N.); jirasak.w@ku.ac.th (J.W.-E.)
[2] Computational Biomodelling Laboratory for Agricultural Science and Technology (CBLAST), Faculty of Science, Kasetsart University, Bangkok 10900, Thailand; nililla.ni@ku.th (N.N.); viwan.jar@nectec.or.th (V.J.); jirasak.w@ku.ac.th (J.W.-E.)
[3] Thailand Center of Excellence in Physics (ThEP Center), Ministry of Higher Education, Science, Research and Innovation, Bangkok 10400, Thailand; nililla.ni@ku.th (N.N.); jirasak.w@ku.ac.th (J.W.-E.)
[4] NSTDA Supercomputer Center (ThaiSC), National Electronics and Computer Technology Center (NECTEC), National Science and Technology Development Agency (NSTDA), Khlong Luang, Pathumthani 12120, Thailand; viwan.jar@nectec.or.th
[5] Department of Chemistry, The University of Western Ontario, 1151 Richmond Street, London, ON N6A 3K7, Canada; mkarttu@uwo.ca
[6] Department of Physics and Astronomy, The University of Western Ontario, 1151 Richmond Street, London, ON N6A 3K7, Canada; mkarttu@uwo.ca
[7] The Centre for Advanced Materials Research (CAMBR), The University of Western Ontario, 1151 Richmond Street, London, ON N6A 3K7, Canada; mkarttu@uwo.ca
* Correspondence: jirasak.w@ku.ac.th

Supporting Information

Table S1. System details. Production simulations were run for 10 µs.

Fullerene concentration	Number of molecules/ions		
	Fullerene	Water	Salt
0%	0	124216	
5%	314	124216	
10%	629	124216	Na^+ (2161)
19%	1296	204216	Cl^- (1158)
20%	1363	124216	
30%	1992	124216	

Table S2. The compositions of lipids in the plasma membrane models.

Phospholipid	Outer leaflet		Inner leaflet	
	Number	Mole fraction (%)	Number	Mole fraction (%)
**Phosphatidylcholine - PC**				
POPC	419	12.12	191	5.96
DOPC	36	1.04	17	0.53
PIPC	617	17.84	281	8.76
PEPC	24	0.69	11	0.34
PAPC	98	2.83	45	1.40
DAPC	12	0.35	5	0.16
PUPC	24	0.69	11	0.34
**Total**	**1230**	35.57	**561**	17.50
**Phosphatidylethanolamine - PE**				
POPE	47	1.36	198	6.18
DOPE	15	0.43	66	2.06
PIPE	31	0.90	132	4.12
PQPE	7	0.20	33	1.03
PAPE	43	1.24	181	5.65
DAPE	27	0.78	115	3.59
PUPE	15	0.43	66	2.06
DUPE	7	0.20	33	1.03
**Total**	**192**	5.55	**824**	25.70

**Sphingomyelin - SM**				
DPSM	212	6.13	97	3.03
DBSM	46	1.33	21	0.66
DXSM	86	2.49	39	1.22
POSM	13	0.38	6	0.19
PGSM	13	0.38	6	0.19
PNSM	132	3.82	60	1.87
BNSM	66	1.91	30	0.94
XNSM	92	2.66	42	1.31
**Total**	**660**	19.09	**301**	9.39
**Phosphatidylserine - PS**				
POPS	0	0.00	69	2.15
PIPS	0	0.00	27	0.84
PQPS	0	0.00	13	0.41
PAPS	0	0.00	160	4.99
DAPS	0	0.00	7	0.22
PUPS	0	0.00	62	1.93
DUPS	0	0.00	7	0.22
**Total**	**0**	0.00	**345**	10.76
**Glycolipid - monosialotetrahexosylganglioside - GM1**				
DPG1	31	0.90	0	0.00
DXG1	17	0.49	0	0.00
PNG1	22	0.64	0	0.00
XNG1	17	0.49	0	0.00
**Total**	**87**	2.52	**0**	0.00
**Glycolipid - monosialodihexosylganglioside - GM3**				
DPG3	31	0.90	0	0.00
DXG3	17	0.49	0	0.00
PNG3	22	0.64	0	0.00
XNG3	17	0.49	0	0.00
**Total**	**87**	2.52	**0**	0.00
**Phosphatidylinositol - PI**				
POPI	0	0.00	47	1.47
PIPI	0	0.00	42	1.31
PAPI	0	0.00	42	1.31
PUPI	0	0.00	17	0.53
**Total**	**0**	0.00	**148**	4.62

**Phosphatidic acid - PA**				
POPA	0	0.00	16	0.50
PIPA	0	0.00	13	0.41
PAPA	0	0.00	13	0.41
PUPA	0	0.00	6	0.19
**Total**	**0**	0.00	**48**	1.50
**Phosphatidylinositol (1-3)phosphates - PIPs**				
POP1	0	0.00	16	0.50
POP2	0	0.00	16	0.50
POP3	0	0.00	16	0.50
**Total**	**0**	0.00	**48**	1.50
**Ceramide - CER**				
DPCE	11	0.32	4	0.12
DXCE	6	0.17	2	0.06
PNCE	7	0.20	3	0.09
XNCE	6	0.17	2	0.06
**Total**	**30**	0.87	**11**	0.34
**Lysophosphatidylcholine - LPC**				
PPC	22	0.64	0	0.00
OPC	7	0.20	0	0.00
IPC	6	0.17	0	0.00
APC	6	0.17	0	0.00
UPC	2	0.06	0	0.00
**Total**	**43**	1.24	**0**	0.00
**Diacylglycerol - DAG**				
PODG	7	0.20	7	0.22
PIDG	6	0.17	7	0.22
PADG	6	0.17	7	0.22
PUDG	2	0.06	2	0.06
**Total**	**21**	0.61	**23**	0.72
**Cholesterol**				
CHOL	**1108**	32.04	**897**	27.98
**All lipid types :**	**3458**	**100**	**3206**	**100**

The plasma membrane lipid compositions were adapted from Table S2 of Ingólfsson et al., 2014[1].

Table S3. Plasma membrane lipid tail distributions.

CG bead type	Acronym	Outer leaflet %	Inner leaflet %	
CCCC	P	21.3	23.8	C16:0-C18:0
CCCCC	B	0.6	0.3	C20:0-C22:0
CCCCCC	X	1.6	0.5	C24:0-C26:0
CCDC	O	7.0	8.6	C16:1-C18:1
CCDCC	G	0.2	0.1	C20:1-C22:1
CCCDCC	N	4.9	1.8	C24:1-C26:1
CDDC	I	8.5	6.5	C18:2
CDDCC	E	0.3	0.1	C20:2
CDDDC	Q	0.1	0.6	C20:3
DDDDC	A	2.5	7.4	C20:4-C22:4
DDDDDC	U	0.6	2.6	C22:5-C22:6
**Total**		**47.7**	**52.3**	

The ratios of lipid tails in both leaflets. For comparison to atomistic lipids, each CG bead represents about four carbon atoms; in the C beads the carbon chain is saturated but the D beads have 1-2 *cis* double bonds[1].

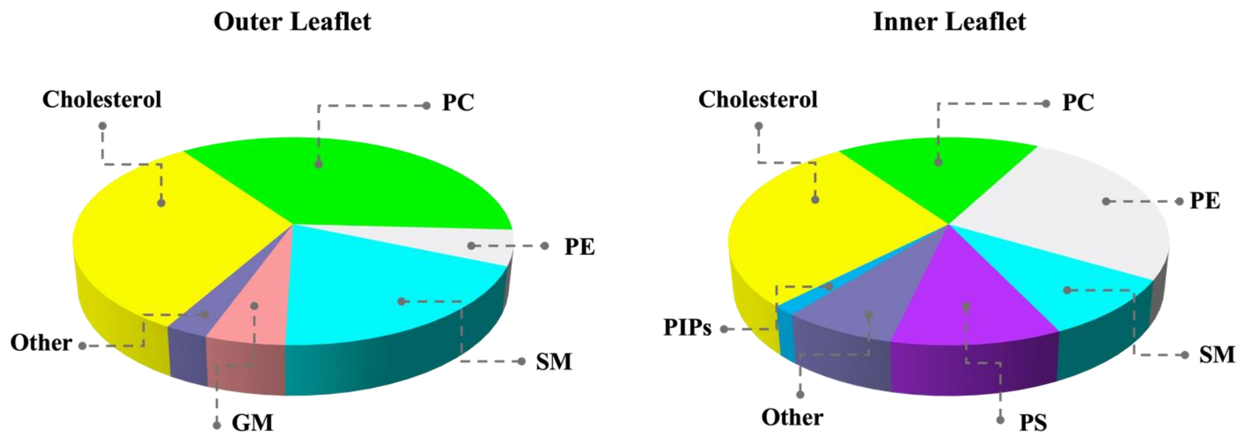

Figure S1. Pie charts showing the distribution of the main lipid headgroups in the plasma membrane. Cholesterol is colored yellow and lipid headgroups are colored by type (PC: green, PE: white, SM: cyan, GM: pink, PS: violet, PIPs: aqua, other: ice blue)

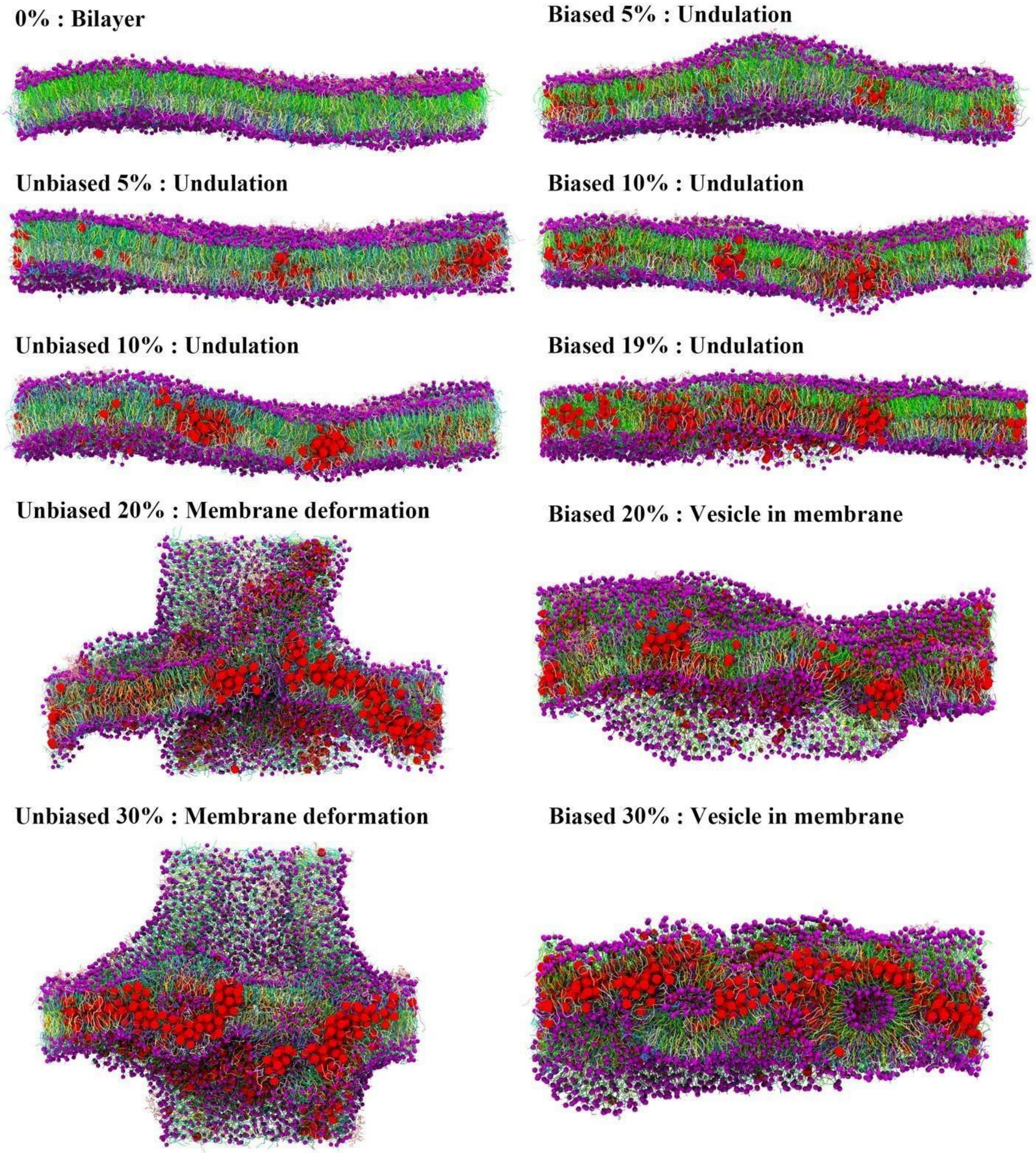

Figure S2. Side views at the end of the simulations at different fullerene concentrations in unbiased and biased simulations. Water molecules and ions are not shown for clarity. Red spheres: fullerenes molecules. Purple spheres: phosphate groups. Lipid colors are shown in Fig. S1.

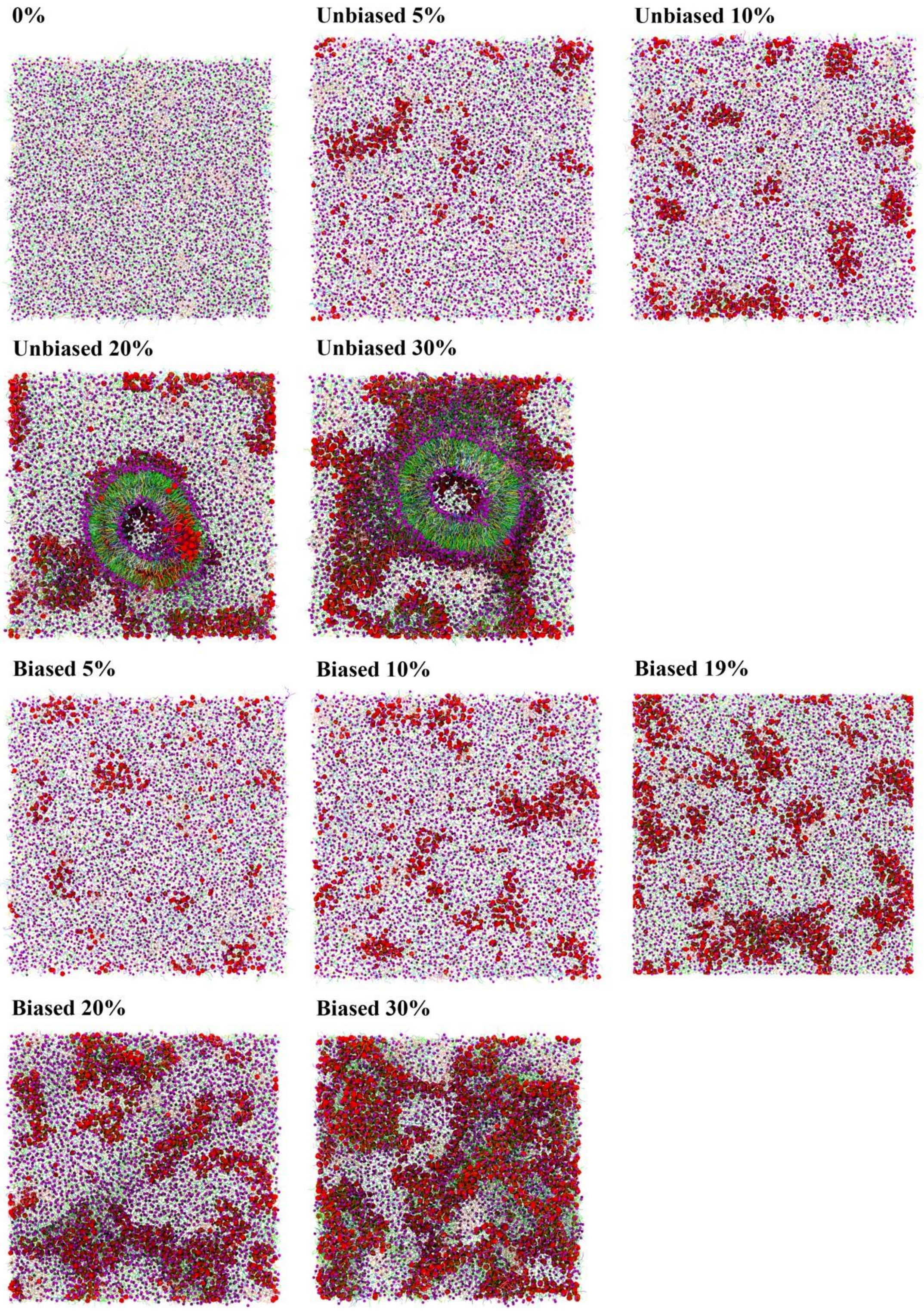

Figure S3. Top views at the end of the simulations at different fullerene concentrations in unbiased and biased simulations of plasma membranes. Water molecules and ions are not shown for clarity. Red spheres: fullerenes molecules. Purple spheres: phosphate groups. Lipid colors are shown in Fig. S1.

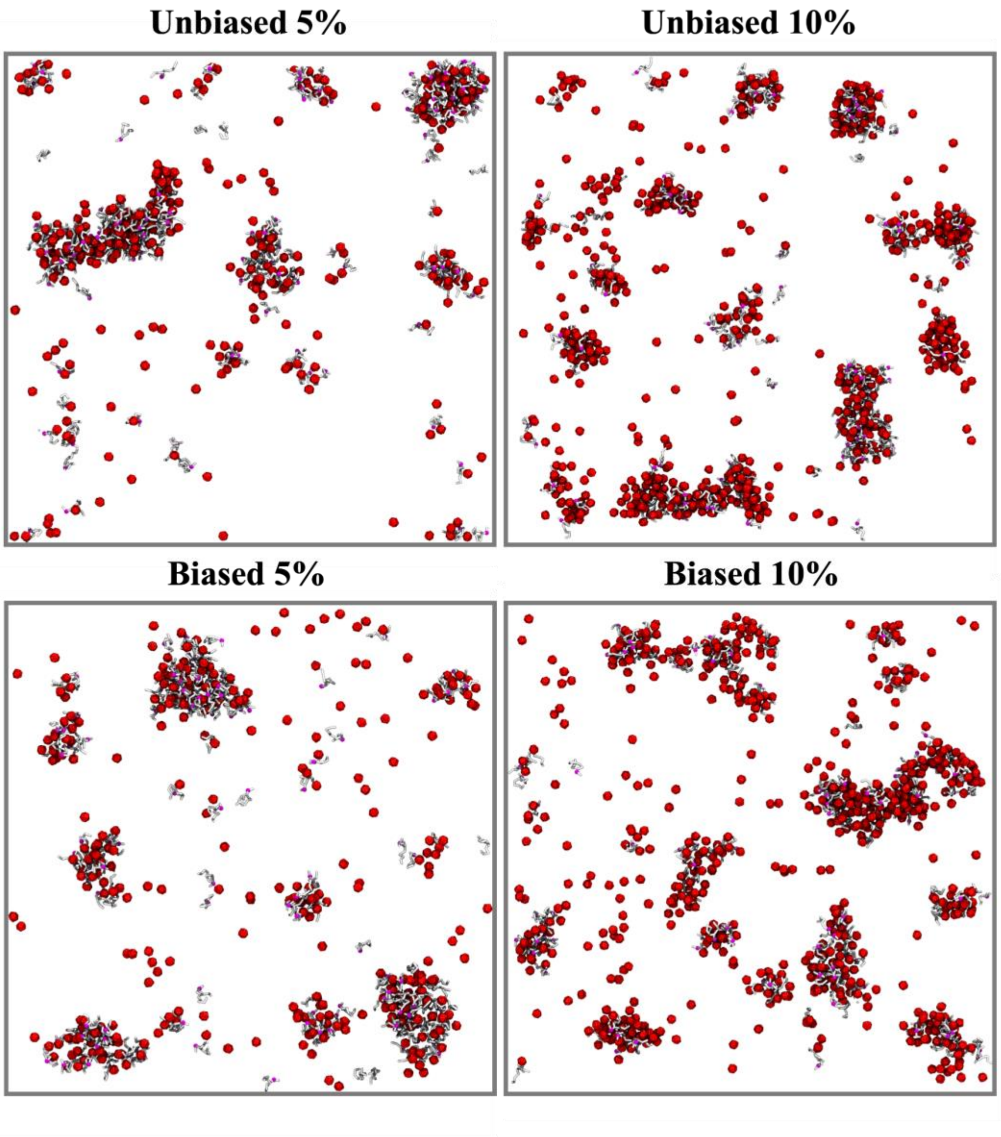

Figure S4. Snapshots illustrating the preference of fullerenes to localize close to the DA tail lipids in biased and unbiased simulations at fullerene concentrations of 5% and 10%. Fullerenes are shown in red, phosphate groups in purple and DA tails in white.

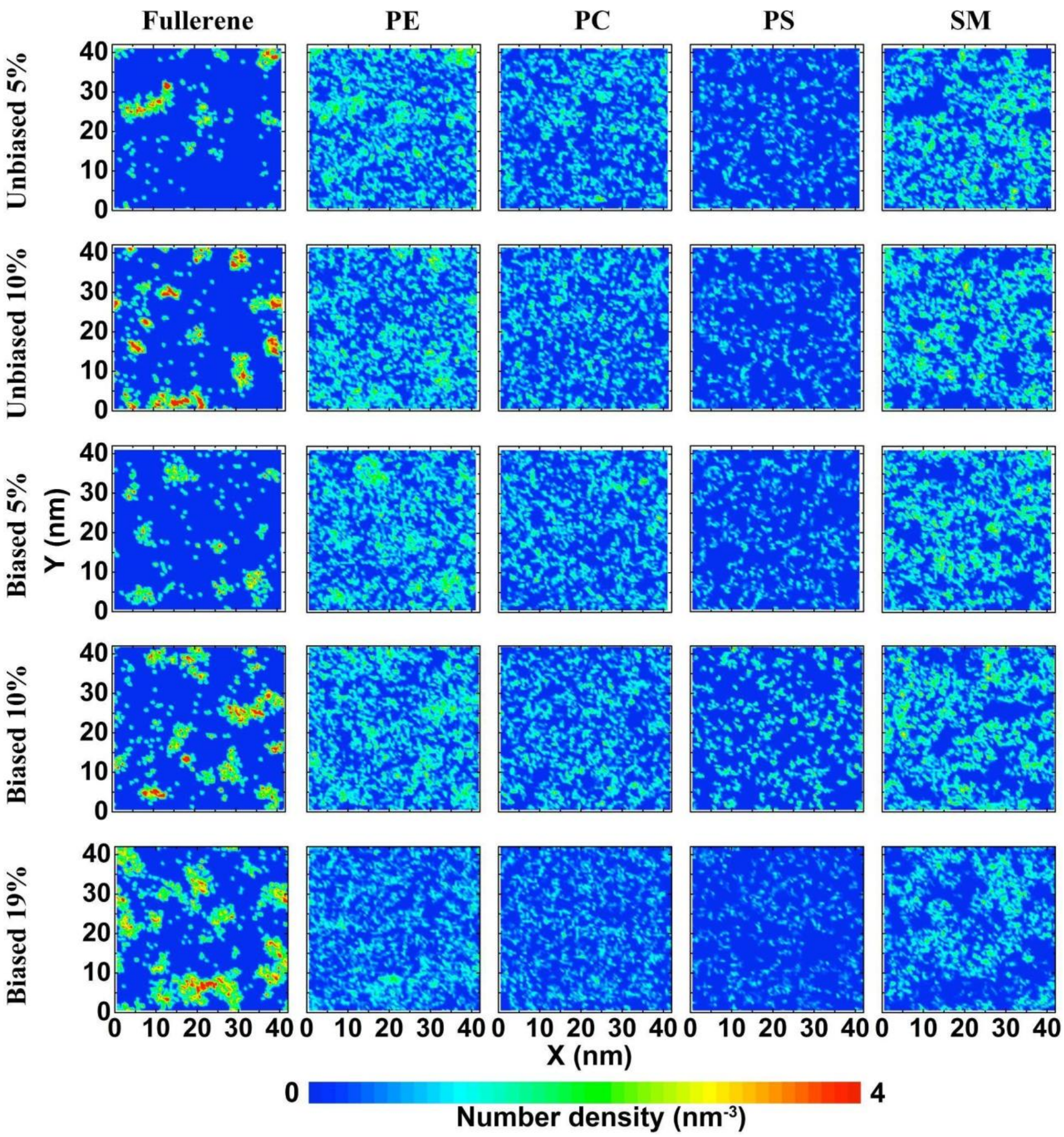

Figure S5. 2D density map of fullerenes, lipid head groups (PE, PC, PS, SM) in biased and unbiased simulations at fullerene concentrations of 5%, 10% and 19%. Each density plot after 10 µs of simulation. The density maps are colored by relative enrichment (red) or depletion (blue).

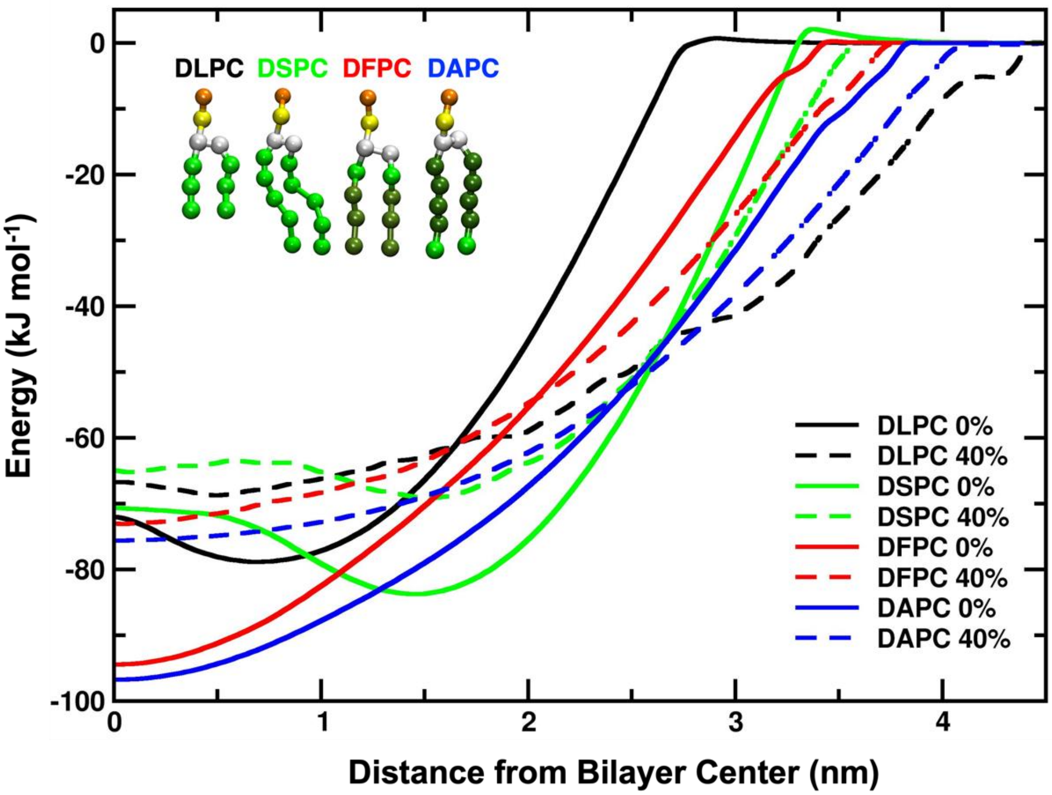

Figure S6. PMF profiles for moving a fullerene across DLPC, DSPC, DFPC and DAPC lipid bilayers at fullerene concentrations of 0% (solid lines) and 40% (dashed lines).

Reference

(1) Ingólfsson, H. I.; Melo, M. N.; van Eerden, F. J.; Arnarez, C.; Lopez, C. A.; Wassenaar, T. A.; Periole, X.; de Vries, A. H.; Tieleman, D. P.; Marrink, S. J. Lipid Organization of the Plasma Membrane. *J. Am. Chem. Soc.* **2014**, *136* (41), 14554–14559. https://doi.org/10.1021/ja507832e.
